# Myricitrin Protects Cardiomyocytes from Hypoxia/Reoxygenation Injury: Involvement of Heat Shock Protein 90

**DOI:** 10.3389/fphar.2017.00353

**Published:** 2017-06-08

**Authors:** Min Wang, Gui-bo Sun, Yu-yang Du, Yu Tian, Ping Liao, Xue-song Liu, Jing-xue Ye, Xiao-bo Sun

**Affiliations:** ^1^Beijing Key Laboratory of Innovative Drug Discovery of Traditional Chinese Medicine (Natural Medicine) and Translational Medicine, Institute of Medicinal Plant Development, Chinese Academy of Medical Sciences–Peking Union Medical CollegeBeijing, China; ^2^College of Pharmacy, Guilin Medical UniversityGuilin, China; ^3^Center of Research and Development on Life Sciences and Environmental Sciences, Harbin University of CommerceHarbin, China

**Keywords:** myricitrin, heart, ischaemia/reperfusion, Hsp90, ROS

## Abstract

Modulation of oxidative stress is therapeutically effective in ischemia/reperfusion (I/R) injury. Myricitrin, a naturally occurring phenolic compound, is a potent antioxidant. However, little is known about its effect on I/R injury to cardiac myocytes. The present study was performed to investigate the potential protective effect of myricitrin against hypoxia/reoxygenation (H/R)-induced H9c2 cardiomyocyte injury and its underlying mechanisms. Myricitrin pretreatment improved cardiomyocyte viability, inhibited ROS generation, maintained the mitochondrial membrane potential, reduced apoptotic cardiomyocytes, decreased the caspase-3 activity, upregulated antiapoptotic proteins and downregulated proapoptotic proteins during H/R injury. Moreover, the potential targets of myricitrin was predicted using Discovery Studio software, and heat shock protein 90 (Hsp90) was identified as the main disease-related target. Further mechanistic investigation revealed that 17-AAG, a pharmacologic inhibitor of Hsp90, significantly blocked the myricitrin-induced cardioprotective effect demonstrated by increased apoptosis and ROS generation. These results suggested that myricitrin provides protection to H9c2 cardiomyocytes against H/R-induced oxidative stress and apoptosis, most likely via increased expression of Hsp90.

## Introduction

Myocardial ischemia/reperfusion (I/R) injury is the leading cause of morbidity and mortality after cardiac surgery and heart infarctions (Sharma et al., 2012). Accumulating evidence from *in vivo* and *in vitro* studies strongly suggests that apoptosis is one of the major mechanisms of cardiomyocyte injury following hypoxia and ischemia–reperfusion (Lam et al., 2013). Reactive oxygen species (ROS) overproduction plays a crucial role in myocyte apoptosis during I/R injury (Takano et al., 2003). Hypoxia/reoxygenation (H/R) initiates the mitochondrial apoptotic pathway by inducing massive ROS production and the loss of mitochondrial membrane potential, which stimulates cytochrome c release and caspase-9 activation, and subsequent caspase-3 activation, ultimately resulting in apoptosis (Ryter et al., 2007). Hence, the inhibition of intracellular ROS levels and prevention of cardiomyocyte apoptosis are effective strategies to ameliorate myocardial I/R injury.

Myricitrin, a naturally occurring flavonoid glycoside belonging to the flavonol subgroup, is abundantly distributed in the fruits, branches, bark, and leaves of *Myrica rubra* and other plants, such as *Myrica esculenta, Ampelopsis grossedentata, Nymphaea lotus*, and *Chrysobalanus icaco* (Huang et al., 2014). It has been shown that myricitrin possesses considerable antioxidant activity (Sun et al., 2013a; Domitrovic et al., 2015), antinociceptive action (Meotti et al., 2007), anti-inflammatory activity (Shimosaki et al., 2011), and anti-fibrotic activity (Geng et al., 2016). We previously demonstrated that myricitrin is able to protect endothelia cells against ROS-induced apoptosis and prevent plaque formation at an early stage in an atherosclerotic mouse model (Sun et al., 2013a; Qin et al., 2015). We also reported that myricitrin inhibits high glucose-induced cardiomyocyte apoptosis to prevent diabetic cardiomyopathy (Zhang et al., 2016). These experiments have indicated that myricitrin can be used for the prevention and treatment of cardiovascular disease. However, there are few reports on the effects and mechanisms of myricitrin on cardiomyocyte apoptosis following I/R.

Therefore, the objective of the present study was to investigate the effects of myricitrin on H9c2 cardiomyocytes subjected to H/R to simulate I/R. The underlying molecular targets of myricitrin against I/R injury were also elucidated using bioinformatics approaches.

## Materials and Methods

### Materials

Myricitrin was provided from the Institute of Medicinal Plant Development (Beijing, China) (Sun et al., 2013a). Cell culture products were purchased from Gibco BRL (Grand island, NY, United States). The fluorescent dye JC-1 was purchased from Sigma-Aldrich (St. Louis, MO, United States). The Annexin V/propidium iodide (PI) apoptosis detection kit was obtained from Invitrogen Corporation (Eugene, OR, United States). Caspase-3 fluorometric assay kits were acquired from BioVision (Milpitas, CA, United States). Primary antibodies against Bcl-2, Bax, Caspase-3, and β-actin were obtained from Santa Cruz Biotechnology, Inc. (Santa Cruz, CA, United States). Primary antibodies against Hsp90, Akt, and p-Akt were purchased from Abcam (Cambridge, United Kingdom). Horseradish peroxidase (HRP)-conjugated secondary antibodies were obtained from CW Biotech (Beijing, China).

### Cell Culture and Hypoxia/Reoxygenation

The H9c2 cardiomyocyte line was obtained from the Chinese Academy of Sciences Cell Bank (Shanghai, China) and cultured as previously described (Wang et al., 2014). Briefly, H9c2 cells were cultured in high glucose DMEM supplemented with 10% (v/v) fetal bovine serum, 1% penicillin/streptomycin (v/v), and 2 mM L-glutamine. The cells were maintained at 37°C with 100% relative humidity in a CO_2_ incubator containing 5% CO_2_.

The H/R procedures were modified from a previous study (Sun et al., 2013b; Wang et al., 2014). The H9c2 cardiomyocytes were cultured in an anaerobic glove box (Coy Laboratory, United States) under hypoxia for 6 h. Subsequently, the cells were removed from the anaerobic glove box and the medium was replaced with high glucose medium and maintained for 12 h in an incubator to mimic reperfusion. For all experiments, the cells were plated at an appropriate density according to the experimental design and grown for 24 h to reach 70 to 80% confluence prior to experimentation.

### Experimental Protocols

The cultured H9c2 cardiomyocytes were randomly divided into different groups. In the control (Con) group, the H9c2 cardiomyocytes were incubated under normoxic conditions for equivalent durations with high glucose DMEM. The H/R group was conducted as described in the preceding section. In the myricitrin-treated group (H/R+Myr), the H9c2 cardiomyocytes were pre-treated with myricitrin for 12 h prior to H/R. The inhibitor-treated groups were processed the same as the H/R+Myr group, but the cells were pre-incubated with 2 μM 17-AAG for 1 h, then removed the medium and treated with myricitrin. The appropriate concentration of the selective HSP90 inhibitor, 17-AAG, was determined on the basis of data found in the literature (Wu et al., 2012) and our preliminary experiments (Supplementary Figure 1).

### Cell Viability Analysis

Cell viability was determined using a 3-(4, 5-dimethylthiazol-2-yl)-2, 5-diphenyl tetrazolium (MTT) assay as previously described (Sun et al., 2012). Briefly, H9c2 cells were plated onto 96-well plates at a density of 1 × 10^4^ cells/well. After designated treatment, 20 μL of MTT (5 mg/mL) was added to each well and incubated for 4 h. The medium was subsequently removed, and the formazan crystals were dissolved in dimethyl sulfoxide (DMSO). The absorbance was read at 570 nm using a microplate reader (TECAN Infinite M1000, Austria).

### Measurement of LDH, CAT, GSH-Px, MDA Levels, and SOD Activity

The supernatant and cells were collected after the different treatments to measure the lactate dehydrogenase (LDH), catalase (CAT), glutathione peroxidase (GSH-Px) and malondialdehyde (MDA) levels as well as superoxide dismutase (SOD) activity using the corresponding detection kits (Nanjing Jiancheng Bioengineering Institute, Nanjing, China) according to the manufacturer’s instructions.

### Detection of Intracellular ROS Production

The effect of myricitrin on the intracellular ROS levels was monitored using the total ROS detection kit according to the manufacturer’s instructions (Enzo Life Sciences, Inc., Farmingdale, NY, United States). Briefly, the cells were preconditioned with myricitrin (40 μM) for 12 h, and further exposed to 6 h of hypoxia followed by 12 h reoxygenation. After treatment, the cells were harvested, placed into 5-mL round-bottom polystyrene tubes, and washed with 1× wash buffer. Subsequently, the cells were centrifuged for 5 min at 400 × *g* at room temperature and the supernatant was discarded. The cells were re-suspended in 500 μL of ROS detection solution, stained in the dark at 37°C for 30 min, and analyzed using flow cytometry (Sun et al., 2011).

### Determination of Mitochondrial Transmembrane Potential (ΔΨm)

We used 5,5′,6,6′-tetrachloro-1,1′,3,3′-tetraethylbenzimidazolyl-carbocyanine iodide (JC-1) (Sigma-Aldrich, St. Louis, MO, United States) to analyze changes in the mitochondrial transmembrane potential as previously described (Sun et al., 2012). The cells were pre-incubated with 40 μM myricitrin for 12 followed by H/R procedure. After treatment, the cells were incubated with 2 μM JC-1 for 30 min in the dark and washed twice with phosphate-buffered saline (PBS). The cells labeled with JC-1 were analyzed by BD FACSCalibur flow cytometry using 488 nm excitation and green (525 nm) or orange-red (575 nm) emission wavelengths with CellQuest software. The JC-1 positive rate was expressed as the percentage of the red/green fluorescence intensity relative to that in the control group.

### Flow Cytometric Detection of Apoptosis

The percentages of early apoptosis and necrosis were measured using an Annexin V-FITC/PI apoptosis kit for flow cytometry according to the manufacturer’s instructions (Invitrogen). After treatment, the cells were harvested and washed twice with cold PBS and subsequently incubated with 5 μL of FITC-Annexin V and 1 μL of the PI working solution (100 μg/mL) in 100 μL of cell suspension for 15 min in the dark at room temperature. Cellular fluorescence was measured by flow cytometry analysis (FACS Calibur^TM^, BD Biosciences, San Jose, CA, United States).

### Analysis of Caspase-9 Activity and Caspase-3 Activation

Caspase-9 activity (Sun et al., 2011) and caspase-3 activation (Sun et al., 2013a) were measured using the corresponding Fluorometric Assay Kits (BioVision, CA, United States) according to the manufacturer’s instructions. To assess caspase-9 activity, the cells were resuspended in lysis buffer and kept on ice for 10 min. Then, 50 ml of 2× reaction buffer containing 10 mM dithiothreitol was added to each sample with 5 ml of 1 mM substrate LEHD-AFC for 1.5 h at 37°C. The samples were determined using a microplate reader (TECAN Infinite M1000, Austria) at the excitation wavelength of 400 nm and the emission wavelength of 505 nm. For analysis of caspase-3 activation, the cells were harvested and incubated with 1 μL substrate FITC-DEVD-FMK for 1 h at 37°C, and then centrifuged at 3000 rpm for 5 min; the supernate was removed. After washing twice with cold PBS, the cells were re-suspended in 100 μL of wash buffer and then transfer the cell suspension to each well in the black microtiter plate. The fluorescence intensity was detected using microplate reader with excitation wavelength of 485 nm and emission wavelength of 535 nm.

### Western Blot Analysis

After treatment, H9c2 cells were harvested, washed with PBS, and lysed with cell lysis buffer containing 1% phenylmethylsulfonyl fluoride. Lysate preparation and Western blot analysis were performed as previously described (Sun et al., 2012). The immunoblots were developed using an electrochemiluminescence (ECL) kit.

### Targets Predicted by Discovery Studio 4.5

The molecular targets of myricitrin were predicted using Discovery Studio (DS) 4.5 (BIOVIA Software, Inc., San Diego, CA, United States). Molecular structure of myricitrin was submitted to Monte Carlo based conformational analysis (Discovery Studio’s FAST algorithm) allowing maximal 255 conformers with less than 20 kcal/mol above the energy minimum. PharmaDB is the only pharmacophore database implemented in DS 4.5. In the profiling with PharmaDB, all pharmacophore models with the shape of the binding pocket were selected for virtual screening using the default settings of the Ligand Profiler module of DS 4.5. The RIGID mode was used as the molecular mapping algorithm. The screening was conducted using the default settings and with a minimal inter-feature distance of 0.5 Å. After inputting all parameters, the job was run, and a result sheet was returned indicating which models were hits and how well myricitrin mapped into each model (fit value), respectively. A higher fit value represents a better fit (Rollinger et al., 2009; Yi et al., 2016).

### Statistical Analyses

The results are expressed as the means ± standard deviation. Comparisons were performed using Student’s *t*-test or one-way ANOVA followed by Tukey’s multiple comparison test using Prism 5.00 software. Statistical significance was set at *p* < 0.05. All data are the result of at least three independent experiments.

## Results

### Myricitrin Alleviated H/R Injury in H9c2 Cells

**FIGURE 1:**
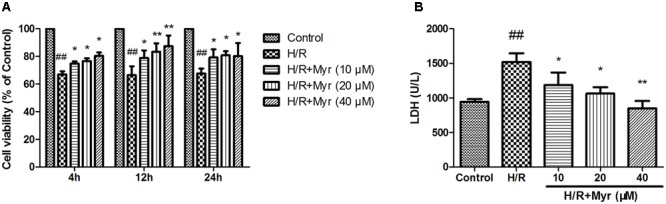
Effects of myricitrin on H/R-induced cell injury in H9c2 cells. **(A)** Pre-treatment of H9c2 cells with various concentrations (0, 10, 20, and 40 μM) of myricitrin for 4, 12, and 24 h, followed by exposure to 6 h of hypoxia and 12 h of reoxygenation. Cell viability was determined using the MTT assay. **(B)** The effect of myricitrin on the level of extracellular LDH was measured using an LDH assay kit. H/R, hypoxia/reoxygenation; Myr, myricitrin. The data are expressed as the means ± SD from three independent experiments. ^##^*p* < 0.01 versus control; ^∗^*p* < 0.05 versus H/R-treated cells; ^∗∗^*p* < 0.01 versus H/R-treated cells.

The protective effect of myricitrin against H/R-induced cell death was detected using an MTT assay. As shown in **Figure 1A** and Supplementary Figure 2, myricitrin pretreatment exhibited a strong protective effect after pretreatment for 12 h. The survival rate increased from 65.48 ± 6.42% (with H/R treatment alone) to 78.92 ± 5.41%, 83.56 ± 5.89%, and 87.48 ± 7.68% after treatment with 10, 20, and 40 μM myricitrin, respectively. LDH, which is secreted from cells after plasma membrane disruption, can be used as an indicator of cell injury. As shown in **Figure 1B**, myricitrin pretreatment decreased the LDH levels in the culture medium in a concentration-dependent manner (*p* < 0.05 or *p* < 0.01). This effect is consistent with its protective effects on cell viability, as assessed using an MTT assay.

### Myricitrin Enhanced Anti-oxidant Enzyme Activity in H/R-Treated H9c2 Cells

**FIGURE 2:**
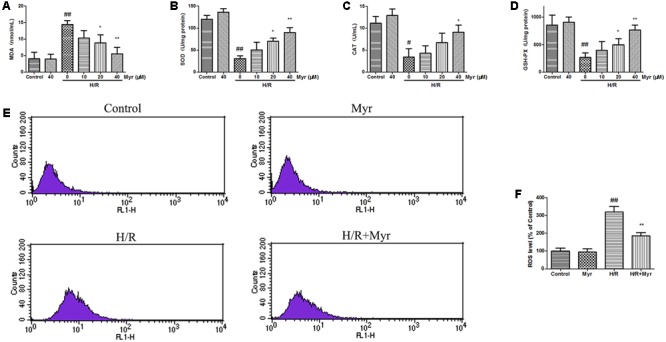
Effects of myricitrin on oxidative damage in H9c2 cells. Cells incubated with different concentrations of myricitrin (10, 20, and 40 μM) for 12 h, were further exposed to 6 h of hypoxia followed by 12 h reoxygenation. **(A)** MDA. **(B)** SOD. **(C)** CAT. **(D)** GSH-Px. **(E)** Flow cytometry analysis of the intracellular ROS. **(F)** Statistical analysis of the flow cytometry data. H/R, hypoxia/reoxygenation; Myr, myricitrin. The data are presented as the means ± SD (*n* = 8 per group). ^#^*p* < 0.05 versus control, ^##^*p* < 0.01 versus control; ^∗^*p* < 0.05 versus H/R-treated cells, ^∗∗^*p* < 0.01 versus H/R-treated cells.

Oxidant stress has long been associated with heart injury in response to I/R (Kalogeris et al., 2012). Membrane lipid oxidation is one of the primary events in oxidative damage, which can be assessed according to the degradation product MDA (Rodrigo et al., 2013). As shown in **Figure 2A**, the H/R group showed a significant increase in intracellular MDA levels, whereas a significant reduction in the levels of MDA was observed in the myricitrin pretreatment group compared with the H/R group. To confirm the antioxidative effects of myricitrin, the activities of the endogenous antioxidative enzymes SOD, CAT, and GSH-Px were detected. Myricitrin pretreatment significantly enhanced the SOD, CAT, and GSH-Px activities compared to the H/R group (**Figures 2B–D**, *p* < 0.05 or *p* < 0.01).

Based on flow cytometry staining, we examined the effect of myricitrin treatment on the intracellular ROS levels. As shown in **Figures 2E,F**, H/R significantly increased the ROS levels by nearly threefold compared with the control, as demonstrated by a shift in fluorescence from left to right. However, myricitrin pretreatment (40 μM) partially blocked this effect, suggesting that myricitrin pretreatment decreased the ROS levels.

### Myricitrin Decreased H/R-Induced Apoptosis in H9c2 Cells

**FIGURE 3:**
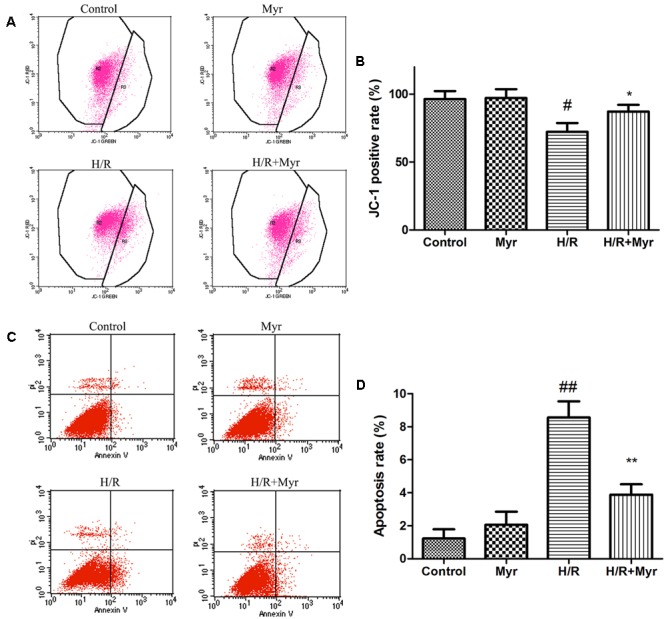
Effects of myricitrin on H/R-induced apoptosis. **(A)** Analysis of JC-1 staining using flow cytometry; **(B)** Statistical analysis of flow cytometry data. **(C)** H9c2 cells stained with FITC-Annexin V-PI measured using flow cytometry; **(D)** Statistical analysis of apoptotic ratios. The data are presented as the means ± SD from three independent experiments. ^#^*p* < 0.05 versus control, ^##^*p* < 0.01 versus control; ^∗^*p* < 0.05 versus H/R-treated cells, ^∗∗^*p* < 0.01 versus H/R-treated cells.

Oxidative stress-induced apoptosis is associated with a loss of mitochondrial membrane potential (ΔΨm), an early marker of apoptosis (Sun et al., 2011). Mitochondrial depolarization was indicated as a decrease in the red/green fluorescence intensity ratio of JC-1 staining. The H/R group exhibited an increase in green fluorescence intensity, indicating ѱm dissipation (**Figures 3A,B**), while pretreatment with myricitrin attenuated ΔΨm depolarization, as indicated by the increased red fluorescence (*p* < 0.05). We further confirmed the anti-apoptotic effect of myricitrin through quantitative analysis of FITC-Annexin V/PI staining using flow cytometry. The percentage of apoptotic cells in the H/R group significantly increased from 1. 23 ± 0.56% to 8.56 ± 0.97% compared with the control group (**Figures 3C,D**). By contrast, pretreatment with myricitrin dramatically decreased the ratio of apoptotic cells to 3.89 ± 0.62%.

### Myricitrin Modulated Apoptosis-Related Protein Activity and Expression in H/R-Treated H9c2 Cells

**FIGURE 4:**
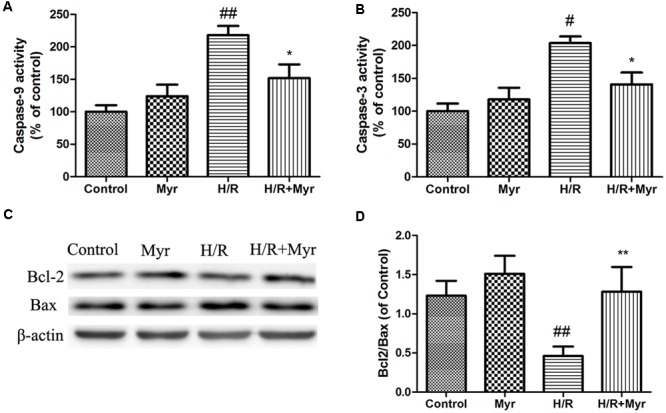
Effects of myricitrin on apoptotic-related protein expression and activity. Proteins related to apoptotic signaling were analyzed using Western blotting. **(A)** Caspase-9 activity. **(B)** Caspase-3 activity. **(C)** Western blot analysis of Bcl-2 and Bax; **(D)** Bcl-2/Bax ratio; β-actin expression was examined as the protein loading control. H/R, hypoxia/reoxygenation; Myr, myricitrin. The data are presented as the means ± SD from three independent experiments. ^#^*p* < 0.05 versus control, ^##^*p* < 0.01 versus control; ^∗^*p* < 0.05 versus H/R-treated cells, ^∗∗^*p* < 0.01 versus H/R-treated cells.

Caspase activity was also used to assess the level of apoptosis (Zhuang et al., 2000). As shown in **Figures 4A,B**, the caspase-9 activity and active caspase-3 levels markedly increased after H/R treatment. Myricitrin pretreatment significantly inhibited these effects (*p* < 0.05). To understand the mechanism of myricitrin treatment on the regulation of cell apoptosis, we measured the effects of myricitrin on the expression of apoptosis-related protein expression using Western blot analysis. As shown in **Figures 4C,D**, the H/R group showed a decrease in expression of the anti-apoptotic protein Bcl-2 and an increase in expression of the pro-apoptotic protein Bax. Pretreatment with myricitrin increased the Bcl-2/Bax expression ratio compared with the H/R group (1. 28 ± 0.31 vs. 0.46 ± 0.12, respectively).

### Target Prediction

**FIGURE 5:**
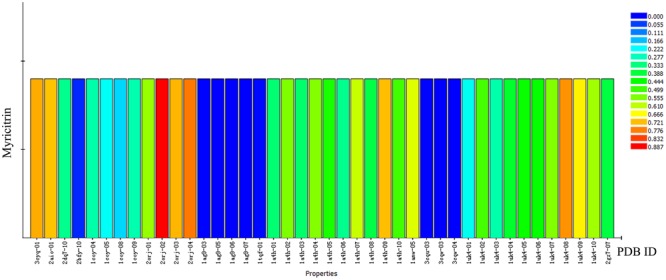
Profiling of the predicted protein targets of myricitrin via Discovery Studio. The *Y*-axis represents the compound myricitrin, the *X*-axis indicates the predicted pharmacophore models (pharmacological targets) of myricitrin. The color from blue to red represents a higher fit value and a better fit.

The molecular targets of myricitrin were predicted using Discovery Studio software (**Figure 5**). The ranking according to fit score in descending order and the top 10 disease-related targets are shown in **Table 1**. Among these targets, we focused on heat shock protein 90 (Hsp90), a critical target in the protection of the myocardium from I/R injury.

**Table 1:** Top ten putative protein targets of myricitrin predicted using Discovery Studio.

Rank	PDB ID	Putative target	Fit value
1	2orj-02	Human surfactant protein D	0.887393
2	2orj-04	Human surfactant protein D	0.781527
3	1uk4-08	SARS Coronavirus main proteinase (3CLpro)	0.760173
4	3cyq-01	C-terminal domain of Helicobacter pylori MotB (residues 125–256)	0.737434
5	2orj-03	Human surfactant protein D	0.72869
6	1a4h-09	ATP/ADP-binding site in the Hsp90	0.720799
7	2aio-01	Metallo-beta-lactamases	0.717487
8	1uk4-09	SARS Coronavirus main proteinase (3CLpro)	0.676778
9	1amw-05	ATP/ADP-binding site in the Hsp90	0.637425
10	1a4h-07	ATP/ADP-binding site in the Hsp90	0.616956

### Hsp90 Contributes to the Cytoprotection of Myricitrin against H/R Injury in H9c2 Cardiomyocytes

**FIGURE 6:**
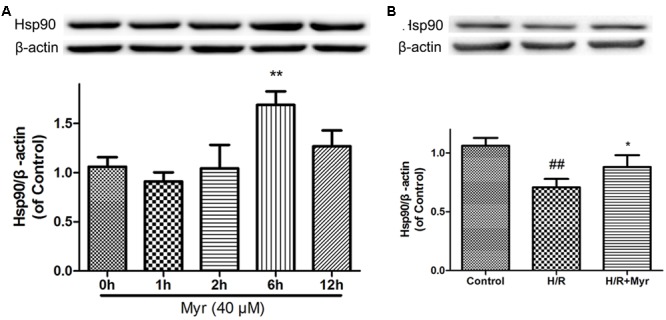
Effects of myricitrin on Hsp90 expression levels. Cell lysates were harvested, and Western blot analysis was performed. **(A)** Effects of myricitrin on Hsp90 expression levels in H9c2 cardiomyocytes. H9c2 cells were treated with myricitrin (40 μM) for various times as indicated. **(B)** Effects of myricitrin on Hsp90 expression levels in H/R-treated cardiomyocytes. Western blot analysis of the Hsp90/β-actin. β-actin expression was examined as the protein loading control. H/R, hypoxia/reoxygenation; Myr, myricitrin. The data are presented as the means ± SD from three independent experiments. ^#^*p* < 0.05 versus control, ^##^*p* < 0.01 versus control; ^∗^*p* < 0.05 versus H/R-treated cells.

Accumulating evidence has shown that upregulation of Hsp90 expression protects cardiomyocytes from I/R-induced damage (Wu et al., 2012). Thus, we detected the expression levels of Hsp90 in myricitrin–treated H9c2 cardiomyocytes. As shown in **Figure 6A**, when H9c2 cells were treated with 40 μM myricitrin for 0–12 h, a significant increase in the expression level of Hsp90 was observed. Next, we detected the effects of myricitrin on the Hsp90 expression levels in H/R-treated cardiomyocytes. **Figure 6B** shows that myricitrin treatment significantly inhibited the downregulation of the H/R-induced Hsp90 expression.

**FIGURE 7:**
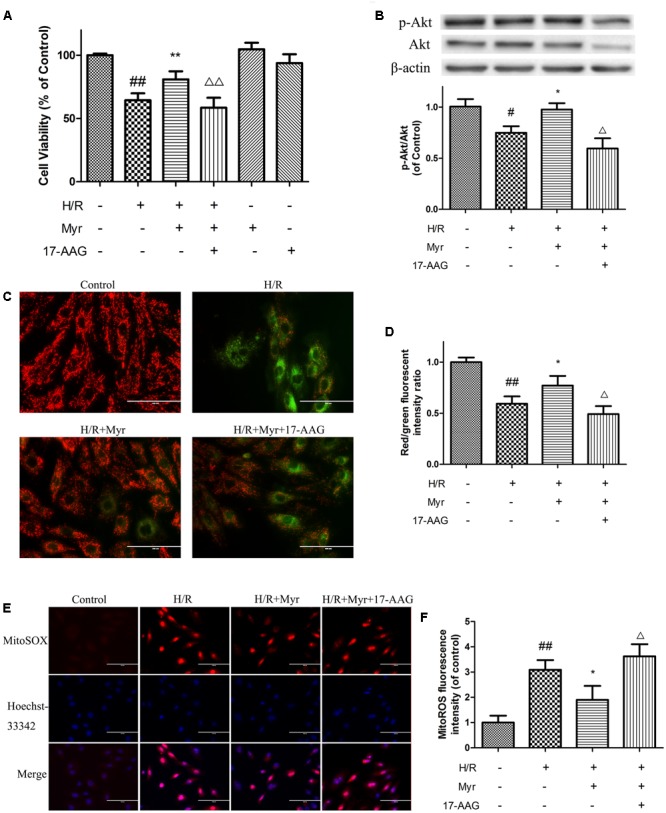
Effects of Hsp90 on the protection of myricitrin against H/R-stimulated cell death and apoptosis. **(A)** Effects of myricitrin and 17-AAG on cell viability in H/R-treated cardiomyocytes. **(B)** Effects of myricitrin and 17-AAG on Akt phosphorylation in H/R-treated cardiomyocytes. Western blotting was performed for each group targeting the ratio of p-Akt to Akt. β-actin expression was examined as the protein loading control. **C** and **D**, effects of myricitrin and 17-AAG on mitochondrial transmembrane permeability transition (ΔΨm) based on JC-1 staining. **(C)** Representative images of JC-1 staining. **(D)** Quantitative analysis of JC-1 staining. **E** and **F**, effects of myricitrin and 17-AAG on mitochondrial ROS levels via MitoSox Red mitochondrial superoxide indicator staining. **(E)** Representative images of mitochondrial ROS staining. **(F)** Quantitative analysis of mitochondrial ROS staining. H/R, hypoxia/reoxygenation; Myr, myricitrin. The data are expressed as the means ± SD from three independent experiments. ^#^*p* < 0.05 versus control; ^##^*p* < 0.01 versus control; ^∗^*p* < 0.05 versus H/R-treated cells; ^∗∗^*p* < 0.01 versus H/R-treated cells; ^Δ^*p* < 0.05 versus H/R+Myr-treated cells, ^ΔΔ^*p* < 0.01 versus H/R+Myr-treated cells.

To determine whether such elevated expression of Hsp90 is responsible for the cell protective effect of myricitrin, a selective inhibitor of Hsp90, 17-AAG, was utilized. As shown in **Figure 7**, the Hsp90 inhibitor 17-AAG reversed the protection against H/R injury by decreased cell viability (**Figure 7A**), phosphorylation levels of the prosurvival protein Akt (**Figure 7B**), and mitochondrial membrane potential (**Figures 7C,D**). In addition, in the presence of 17-AAG, the decreased mitochondrial ROS levels induced by myricitrin were also abolished (**Figures 7E,F**). These results suggest that Hsp90 is involved in the protective effect of myricitrin against H/R injury in H9c2 cells.

## Discussion

Oxidative stress has been implicated in injury associated with myocardial ischemia/reperfusion through induced ROS production (Kalogeris et al., 2012). Flavonoids, which contain a catechol group, have been described as scavengers and mimics of SOD and play an important role in oxidative stress (Meotti et al., 2006). Myricitrin, belonging to the flavonol subgroup, reportedly possesses effective antioxidant activity (Domitrovic et al., 2015), with stronger free radical scavenging activity than other flavonol rhamnosides or quercetin (Wu et al., 2008). Similarly, the results of the present study clearly showed that ROS production was well preserved as a result of myricitrin pretreatment during H/R; this effect was accompanied by significantly elevated activities of SOD, GSH-Px, and CAT and decreased levels of MDA, suggesting that the cardioprotective effects of myricitrin against I/R are largely associated with its anti-oxidative capacity.

In addition to producing oxidative injury, cardiomyocyte apoptosis is also one of the major contributors to the development of myocardial I/R injury (van Empel et al., 2005). Hypoxia and excess ROS alter the mitochondrial structure and trigger mitochondrial depolarization as well as dissipation of ΔΨm (Sinha et al., 2013). Disruption of ΔΨm induces the release of proapoptotic molecules from the mitochondria and subsequently activates caspases, resulting in apoptosis (Sinha et al., 2013). In the present study, the results indicated that myricitrin maintained the mitochondrial membrane potential, decreased caspase-9 activity and caspase-3 activation, and reduced the apoptosis rate of the H9c2 cardiomyocytes against H/R. Caspase-3 activity can be induced by proapoptotic Bax and inhibited by anti-apoptotic Bcl-2 (Kaushal et al., 2004). Western blot analysis showed that myricitrin increased the Bcl-2/Bax ratio compared with the H/R group. These data demonstrated that myricitrin effectively inhibits H/R-induced apoptosis and provides further evidence supporting its anti-apoptotic role (Qin et al., 2015; Zhang et al., 2016).

Myricitrin interacts with proteins, such as PI-3 kinase, nitric oxide synthase (NOS), PKCα, and PKC𝜀 (Pereira et al., 2011; Schwanke et al., 2013; Qin et al., 2015). In addition, previous studies have demonstrated that myricitrin potently inhibits calcium transport, likely through the interaction of the phenolic groups of this flavonoid with the Ca^2+^ transporting protein (Thiyagarajah et al.1991; Meotti et al., 2007). In the present study, we investigated the potential targets that may have significant relationships with the pharmacologic effects of myricitrin using bioinformatics approaches. Among the targets of myricitrin, the main protein involved in I/R injury is HSP90. Hsp90, as an abundant chaperone that regulates cell signaling and the functions of many proteins that bind to it, referred to as “client proteins” (Wang et al., 2009). According to previous studies, Hsp90 is a direct effector that enables the activation of the prosurvival PI3K/Akt pathway (Barksdale and Bijur, 2009). In recent years, studies have shown that Hsp90 mediates the mitochondrial import of PKC𝜀 (Budas et al., 2010; Zhong et al., 2014) and plays an integral role in endothelial NOS signaling (Amour et al., 2009), thereby efficiently protecting the myocardium against I/R injury (Kupatt et al., 2004; Zhu et al., 2016). Furthermore, Hsp90 facilitates the inhibitory effect of HAX-1 on SR calcium uptake, which is protective in I/R injury (Lam et al., 2013). Thus, we speculated that Hsp90 is one of the targets of myricitrin to relieve the H/R injury of cardiomyocytes.

Next, we further investigated the roles of Hsp90 in the cardioprotection of myricitrin against H/R injury. As expected, myricitrin pretreatment obviously blocked the inhibitory effect of H/R on the expression of Hsp90. Hsp90 acts as an upstream regulator of the serine/threonine protein kinase Akt survival pathway during cellular stress (Barksdale and Bijur, 2009). We previously showed that myricitrin mediates cardiovascular protection through activation of the Akt signaling pathway (Qin et al., 2015; Zhang et al., 2016). In the present study, the addition of the Hsp90 inhibitor 17-AAG substantially decreased Akt phosphorylation induced by myricitrin, thereby attenuating the prosurvival effects of the PI3-K/Akt pathway. In addition, inhibition of Hsp90 by 17-AAG markedly abolished myricitrin-induced cardioprotection against H/R injury, as shown by the increased overproduction of mitochondrial ROS and loss of MMP. These findings are consistent with recent reports that Hsp90 is anti-apoptotic in cardiomyocytes through antioxidation and the preservation of mitochondrial function under stress conditions (Wu et al., 2012). Based on these results, we suggest that Hsp90 is involved in the cardio-protective effects of myricitrin and that the interaction between myricitrin and Hsp90 may promote the anti-apoptotic PI3-K/Akt signaling pathway in cardiomyocytes during H/R injury.

## Conclusion

The results of the present study indicated that myricitrin, a dietary flavonoid compound, exerts a profound cardioprotective effect against H/R injury for H9c2 cardiomyocytes through suppression of oxidative stress and apoptosis and that Hsp90 contributes to the myricitrin-induced cardioprotective effect, at least in part, through the PI3-K/Akt pathway. Although more experiments are required to elucidate the precise mechanisms underlying this protection because HSP90 associates with a variety of other signaling molecules, these results suggest that myricitrin may be a promising therapeutic candidate for the treatment of myocardial I/R injury.

## Author Contributions

G-bS, J-xY, and X-bS conducted the study. MW designed the detailed experiments, performed the study, and collected and analyzed data. Y-yD, YT, PL, and X-sL took part in the cell experiments in this study. All Authors commented the study and approved the final manuscript.

## Conflict of Interest Statement

The authors declare that the research was conducted in the absence of any commercial or financial relationships that could be construed as a potential conflict of interest.

## References

[B1] Amour J. Brzezinska A. K. Weihrauch D. Billstrom A. R. Zielonka J. Krolikowski J. G. (2009). Role of heat shock protein 90 and endothelial nitric oxide synthase during early anesthetic and ischemic preconditioning. *Anesthesiology* 110 317–325. 10.1097/ALN.0b013e3181942cb4 19194158 PMC2730207

[B2] Barksdale K. A. Bijur G. N. (2009). The basal flux of Akt in the mitochondria is mediated by heat shock protein 90. *J. Neurochem.* 108 1289–1299. 10.1111/j.1471-4159.2009.05878.x 19187436 PMC2696161

[B3] Budas G. R. Churchill E. N. Disatnik M. H. Sun L. Mochly-Rosen D. (2010). Mitochondrial import of PKCepsilon is mediated by HSP90: a role in cardioprotection from ischaemia and reperfusion injury. *Cardiovasc. Res.* 88 83–92. 10.1093/cvr/cvq154 20558438 PMC2936125

[B4] Domitrovic R. Rashed K. Cvijanovic O. Vladimir-Knezevic S. Skoda M. Visnic A. (2015). Myricitrin exhibits antioxidant, anti-inflammatory and antifibrotic activity in carbon tetrachloride-intoxicated mice. *Chem. Biol. Interact.* 230 21–29. 10.1016/j.cbi.2015.01.030 25656916

[B5] Geng Y. Sun Q. Li W. Lu Z. M. Xu H. Y. Shi J. S. (2016). The common dietary flavonoid myricetin attenuates liver fibrosis in carbon tetrachloride-treated mice. *Mol. Nutr. Food Res.* 61:1600392 10.1002/mnfr.201600392 27983763

[B6] Huang Q. Gao B. Wang L. Hu Y. Q. Lu W. G. Yang L. (2014). Protective effects of myricitrin against osteoporosis via reducing reactive oxygen species and bone-resorbing cytokines. *Toxicol. Appl. Pharmacol.* 280 550–560. 10.1016/j.taap.2014.08.004 25130202

[B7] Kalogeris T. Baines C. P. Krenz M. Korthuis R. J. (2012). Cell biology of ischemia/reperfusion injury. *Int. Rev. Cell Mol. Biol.* 298 229–317. 10.1016/B978-0-12-394309-5.00006-7 22878108 PMC3904795

[B8] Kaushal G. P. Liu L. Kaushal V. Hong X. Melnyk O. Seth R. (2004). Regulation of caspase-3 and -9 activation in oxidant stress to RTE by forkhead transcription factors, Bcl-2 proteins, and MAP kinases. *Am. J. Physiol. Renal Physiol.* 287 F1258–F1268. 10.1152/ajprenal.00391.2003 15304372

[B9] Kupatt C. Dessy C. Hinkel R. Raake P. Daneau G. Bouzin C. (2004). Heat shock protein 90 transfection reduces ischemia-reperfusion-induced myocardial dysfunction via reciprocal endothelial NO synthase serine 1177 phosphorylation and threonine 495 dephosphorylation. *Arterioscler. Thromb. Vasc. Biol.* 24 1435–1441. 10.1161/01.ATV.0000134300.87476.d1 15178564

[B10] Lam C. K. Zhao W. Cai W. Vafiadaki E. Florea S. M. Ren X. (2013). Novel role of HAX-1 in ischemic injury protection involvement of heat shock protein 90. *Circ. Res.* 112 79–89. 10.1161/CIRCRESAHA.112.279935 22982986 PMC3537902

[B11] Meotti F. C. Fachinetto R. Maffi L. C. Missau F. C. Pizzolatti M. G. Rocha J. B. (2007). Antinociceptive action of myricitrin: involvement of the K+ and Ca2+ channels. *Eur. J. Pharmacol.* 567 198–205. 17467689 10.1016/j.ejphar.2007.03.039

[B12] Meotti F. C. Missau F. C. Ferreira J. Pizzolatti M. G. Mizuzaki C. Nogueira C. W. (2006). Anti-allodynic property of flavonoid myricitrin in models of persistent inflammatory and neuropathic pain in mice. *Biochem. Pharmacol.* 72 1707–1713. 17070780 10.1016/j.bcp.2006.08.028

[B13] Pereira M. Siba I. P. Chioca L. R. Correia D. Vital M. A. Pizzolatti M. G. (2011). Myricitrin, a nitric oxide and protein kinase C inhibitor, exerts antipsychotic-like effects in animal models. *Prog. Neuropsychopharmacol. Biol. Psychiatry* 35 1636–1644. 10.1016/j.pnpbp.2011.06.002 21689712

[B14] Qin M. Luo Y. Meng X. B. Wang M. Wang H. W. Song S. Y. (2015). Myricitrin attenuates endothelial cell apoptosis to prevent atherosclerosis: an insight into PI3K/Akt activation and STAT3 signaling pathways. *Vascul. Pharmacol.* 70 23–34. 10.1016/j.vph.2015.03.002 25849952

[B15] Rodrigo R. Libuy M. Feliu F. Hasson D. (2013). Oxidative stress-related biomarkers in essential hypertension and ischemia-reperfusion myocardial damage. *Dis. Markers* 35 773–790. 10.1155/2013/974358 24347798 PMC3856219

[B16] Rollinger J. M. Schuster D. Danzl B. Schwaiger S. Markt P. Schmidtke M. (2009). In silico target fishing for rationalized ligand discovery exemplified on constituents of *Ruta graveolens*. *Planta Med.* 75 195–204. 10.1055/s-0028-1088397 19096995 PMC3525952

[B17] Ryter S. W. Kim H. P. Hoetzel A. Park J. W. Nakahira K. Wang X. (2007). Mechanisms of cell death in oxidative stress. *Antioxid. Redox. Signal.* 9 49–89. 10.1089/ars.2007.9.49 17115887

[B18] Schwanke R. C. Marcon R. Meotti F. C. Bento A. F. Dutra R. C. Pizzollatti M. G. (2013). Oral administration of the flavonoid myricitrin prevents dextran sulfate sodium-induced experimental colitis in mice through modulation of PI3K/Akt signaling pathway. *Mol. Nutr. Food Res.* 57 1938–1949. 10.1002/mnfr.201300134 23861337

[B19] Sharma V. Bell R. M. Yellon D. M. (2012). Targeting reperfusion injury in acute myocardial infarction: a review of reperfusion injury pharmacotherapy. *Expert Opin. Pharmacother.* 13 1153–1175. 10.1517/14656566.2012.685163 22594845

[B20] Shimosaki S. Tsurunaga Y. Itamura H. Nakamura M. (2011). Anti-allergic effect of the flavonoid myricitrin from *Myrica rubra* leaf extracts in vitro and in vivo. *Nat. Prod. Res.* 25 374–380. 10.1080/14786411003774320 21328132

[B21] Sinha K. Das J. Pal P. B. Sil P. C. (2013). Oxidative stress: the mitochondria-dependent and mitochondria-independent pathways of apoptosis. *Arch. Toxicol.* 87 1157–1180. 10.1007/s00204-013-1034-4 23543009

[B22] Sun G. B. Qin M. Ye J. X. Pan R. L. Meng X. B. Wang M. (2013a). Inhibitory effects of myricitrin on oxidative stress-induced endothelial damage and early atherosclerosis in ApoE-/- mice. *Toxicol. Appl. Pharmacol.* 271 114–126. 10.1016/j.taap.2013.04.015 23639522

[B23] Sun J. Sun G. Meng X. Wang H. Wang M. Qin M. (2013b). Ginsenoside RK3 Prevents hypoxia-reoxygenation induced apoptosis in H9c2 cardiomyocytes via AKT and MAPK pathway. *Evid. Based Complement Alternat. Med.* 690190:27 10.1155/2013/690190 PMC3712237 23935671

[B24] Sun G. B. Sun X. Wang M. Ye J. X. Si J. Y. Xu H. B. (2012). Oxidative stress suppression by luteolin-induced heme oxygenase-1 expression. *Toxicol. Appl. Pharmacol.* 265 229–240. 10.1016/j.taap.2012.10.002 23051850

[B25] Sun X. Sun G. B. Wang M. Xiao J. Sun X. B. (2011). Protective effects of cynaroside against H(2)O(2)-induced apoptosis in H9c2 cardiomyoblasts. *J. Cell. Biochem.* 112 2019–2029. 10.1002/jcb.23121 21445859

[B26] Takano H. Zou Y. Hasegawa H. Akazawa H. Nagai T. Komuro I. (2003). Oxidative stress-induced signal transduction pathways in cardiac myocytes: involvement of ROS in heart diseases. *Antioxid. Redox. Signal.* 5 789–794. 10.1089/152308603770380098 14588152

[B27] Thiyagarajah P. Kuttan S. C. Lim S. C. Teo T. S. Das N. P. (1991). Effect of myricetin and other flavonoids on the liver plasma membrane Ca2+ pump. Kinetics and structure-function relationships. *Biochem. Pharmacol.* 41 669–675. 1998524 10.1016/0006-2952(91)90065-d

[B28] van Empel V. P. Bertrand A. T. Hofstra L. Crijns H. J. Doevendans P. A. De Windt L. J. (2005). Myocyte apoptosis in heart failure. *Cardiovasc. Res.* 67 21–29. 15896727 10.1016/j.cardiores.2005.04.012

[B29] Wang M. Meng X. B. Yu Y. L. Sun G. B. Xu X. D. Zhang X. P. (2014). Elatoside C protects against hypoxia/reoxygenation-induced apoptosis in H9c2 cardiomyocytes through the reduction of endoplasmic reticulum stress partially depending on STAT3 activation. *Apoptosis* 19 1727–1735. 10.1007/s10495-014-1039-3 25326083

[B30] Wang W. Peng Y. Wang Y. Zhao X. Yuan Z. (2009). Anti-apoptotic effect of heat shock protein 90 on hypoxia-mediated cardiomyocyte damage is mediated via the phosphatidylinositol 3-kinase/AKT pathway. *Clin. Exp. Pharmacol. Physiol.* 36 899–903. 10.1111/j.1440-1681.2009.05167.x 19298537

[B31] Wu J. H. Huang C. Y. Tung Y. T. Chang S. T. (2008). Online RP-HPLC-DPPH screening method for detection of radical-scavenging phytochemicals from flowers of *Acacia confusa*. *J. Agric. Food Chem.* 56 328–332. 10.1021/jf072314c 18163556

[B32] Wu K. Xu W. You Q. Guo R. Feng J. Zhang C. (2012). Increased expression of heat shock protein 90 under chemical hypoxic conditions protects cardiomyocytes against injury induced by serum and glucose deprivation. *Int. J. Mol. Med.* 30 1138–1144. 10.3892/ijmm.2012.1099 22922826

[B33] Yi F. Tan X. L. Yan X. Liu H. B. (2016). In silico profiling for secondary metabolites from Lepidium meyenii (maca) by the pharmacophore and ligand-shape-based joint approach. *Chin Med.* 11:42 10.1186/s13020-016-0112-y PMC5037646 27708692

[B34] Zhang B. Chen Y. Shen Q. Liu G. Ye J. Sun G. (2016). Myricitrin attenuates high glucose-induced apoptosis through activating Akt-Nrf2 signaling in H9c2 cardiomyocytes. *Molecules* 21:E880 10.3390/molecules21070880 PMC6274128 27399653

[B35] Zhong G. Q. Tu R. H. Zeng Z. Y. Li Q. J. He Y. Li S. (2014). Novel functional role of heat shock protein 90 in protein kinase C-mediated ischemic postconditioning. *J. Surg. Res.* 189 198–206. 10.1016/j.jss.2014.01.038 24742623

[B36] Zhu W. S. Guo W. Zhu J. N. Tang C. M. Fu Y. H. Lin Q. X. (2016). Hsp90aa1: a novel target gene of miR-1 in cardiac ischemia/reperfusion injury. *Sci. Rep.* 6:24498 10.1038/srep24498 PMC4830926 27076094

[B37] Zhuang S. Demirs J. T. Kochevar I. E. (2000). p38 mitogen-activated protein kinase mediates bid cleavage, mitochondrial dysfunction, and caspase-3 activation during apoptosis induced by singlet oxygen but not by hydrogen peroxide. *J. Biol. Chem.* 275 25939–25948. 10837470 10.1074/jbc.M001185200

