# Supplementary material for: Myricitrin Protects Cardiomyocytes from Hypoxia/Reoxygenation Injury: Involvement of Heat Shock Protein 90
Source: Front Pharmacol. 2017 Jun 8;8:353. doi: 10.3389/fphar.2017.00353 (PMC5462924; doi:10.3389/fphar.2017.00353)
Supplement: Supplementary file 1 [file Image_1.PDF]

Supplementary Material

Myricitrin protects cardiomyocytes from hypoxia/reoxygenation injury: Involvement of heat shock protein 90

Min Wang[1], Gui-bo Sun[1,*], Yu-yang Du[1], Yu Tian[1], Ping Liao[2], Xue-song Liu[3], Jing-xue Ye[1,*], and Xiao-bo Sun[1,*]

[1]Beijing Key Laboratory of Innovative Drug Discovery of Traditional Chinese Medicine (Natural Medicine) and Translational Medicine, Institute of Medicinal Plant Development, Chinese Academy of Medical Sciences & Peking Union Medical College, Beijing, 100193, P. R. China.

[2]College of Pharmacy, Guilin Medical University, Guilin 541000, China.

[3]Harbin University of Commerce, Harbin, 150076, Heilongjiang, P. R. China.

*** Correspondence:** Gui-bo Sun: gbsun@implad.ac.cn, Jing-xue Ye: yejingxue2002@126.com, Xiao-bo Sun: sunsubmit@163.com.

Supplementary Figures

Supplementary Figure 1

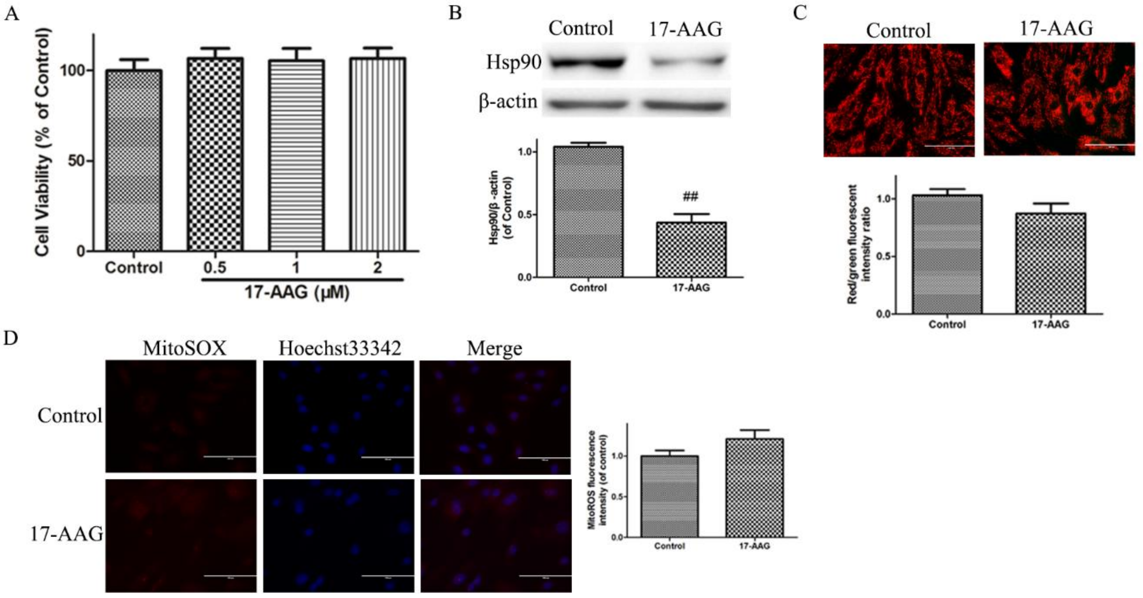

Supplementary Figure 1. The effects of 17-AAG on normal H9c2 cardiomyocytes. (A) Cell viability was determined using an MTT assay. (B) Western blot analysis of Hsp90; β-actin expression was examined as the protein loading control. (C) Effects of 17-AAG on mitochondrial transmembrane permeability transition (ΔΨm) based on JC-1 staining. (D) Effects of 17-AAG on mitochondrial ROS levels via MitoSoxRed mitochondrial superoxide indicator staining. H/R, Hypoxia/Reoxygenation; Myr, myricitrin. The data are presented as the means±SD from three independent experiments. ## *p*<0.01 versus control.

Supplementary Figure 2

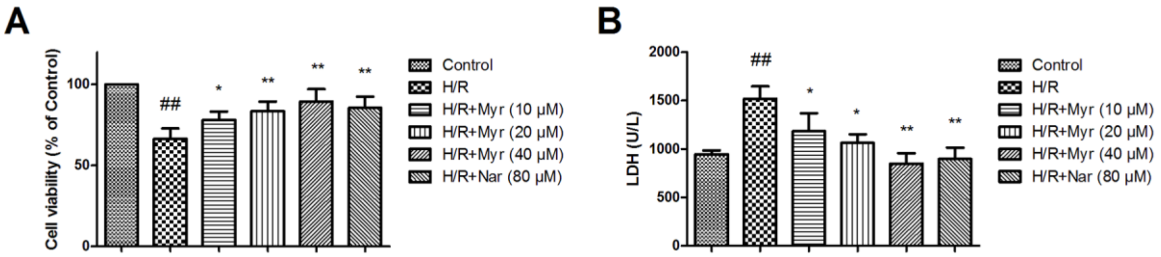

Supplementary Figure 2. Effects of myricitrin on H/R-induced cell injury in H9c2 cells. (A) Pre-treatment of H9c2 cells with the indicated concentrations of myricitrin for 12 h, followed by exposure to 6 h of hypoxia and 12 h of reoxygenation. Naringin (80 μM) as a positive control was pretreated for 6 h followed by H/R. Cell viability was determined using the MTT assay. (B) The effect of myricitrin on the level of extracellular LDH was measured using an LDH assay kit. H/R, Hypoxia/Reoxygenation; Myr, myricitrin. The data are expressed as the means ± SD from three independent experiments. ##P < 0.01 versus control; *P < 0.05 versus H/R-treated cells; **P < 0.01 versus H/R-treated cells.
